# The Benefits of Early Book Sharing (BEBS) for child cognitive and socio-emotional development in South Africa: study protocol for a randomised controlled trial

**DOI:** 10.1186/s13063-017-1790-1

**Published:** 2017-03-09

**Authors:** Nicholas Dowdall, Peter J. Cooper, Mark Tomlinson, Sarah Skeen, Frances Gardner, Lynne Murray

**Affiliations:** 1 Department of Social Policy and Intervention, Oxford University, Oxford, UK; 2 University of Reading, Reading, UK; 3 University of Cape Town, Cape Town, South Africa; 4 Stellenbosch University, Stellenbosch, South Africa

**Keywords:** Early childhood development, Parenting intervention, Book-sharing, South Africa, Violence prevention

## Abstract

**Background:**

Children in low and middle-income countries (LMICs) are at risk for problems in their cognitive, social and behavioural development. Factors such as a lack of cognitive stimulation, harsh parenting practices, and severe and persistent aggression in early childhood are central to the genesis of these problems. Interventions that target the intersection between early childhood development, parenting, and early violence prevention are required in order to meaningfully address these problems.

**Methods:**

We are conducting a randomised controlled trial to evaluate a parenting intervention for caregivers of children aged between 23 and 27 months, designed to promote child cognitive and socioemotional development in Khayelitsha, a low-income peri-urban township in South Africa. Families are randomly allocated to a book-sharing intervention group or to a wait-list control group. In the intervention, we train caregivers in supportive book-sharing with young children. Training is carried out in small groups over a period of 8 weeks. Data are collected at baseline, post intervention and at 6 months post intervention. In addition to targeting child cognitive development, the intervention aims to improve child socioemotional functioning.

**Discussion:**

The Benefits of Early Book Sharing (BEBS) trial aims to evaluate the impact of an early parenting intervention on several key risk factors for the development of violence, including aspects of parenting and child cognition, prosocial behaviour, aggression, and socioemotional functioning. The study is being carried out in a LMIC where violence constitutes a major social and health burden. Since the intervention is brief and, with modest levels of training, readily deliverable in LMIC contexts, a demonstration that it is of benefit to both child cognitive and socioemotional development would be of significance.

**Trial registration:**

The BEBS trial is registered on the International Standard Randomised Controlled Trial Number database, registration number ISRCTN71109104. Registered on 9 February 2016. This is version 1 of the protocol for the BEBS trial.

**Electronic supplementary material:**

The online version of this article (doi:10.1186/s13063-017-1790-1) contains supplementary material, which is available to authorized users.

## Background

### Child development problems in low and middle-income countries (LMICs)

Children in low and middle-income countries (LMICs) are at risk for problems in their cognitive, social, and behavioural development [1]. These risks carry not only a considerable social burden, but also have a major adverse financial impact on affected societies [2]. These children’s problems commonly occur in the context of widespread poverty. Indeed, conservative estimates suggest that in excess of 200 million children, primarily in sub-Saharan Africa and Asia, are failing to reach their developmental potential as a result of such poverty [1]. What makes optimal development so difficult for young children to achieve in these contexts is a combination of adverse factors that impair cognitive, social and emotional development, as well as physical growth and health [3]. These predominantly occur within a context of severe community stress, often characterised by high levels of aggression and violent crime, and lack of opportunities for escape from disadvantage. In this context, caregivers who themselves have been educationally and socially disadvantaged are likely to replicate childrearing and relationship practices that contribute to cognitive and socioemotional deficits and behaviour problems [3].

### Cognitive development

A large body of research from high-income countries (HICs) has shown that early cognitive development is a critical determinant of subsequent school progress and literacy [4–6]. Evidence suggests that a similar relationship obtains in LMIC settings [7–9]. Hence, the early developmental deficits so prevalent in LMIC contexts are likely to play a critical role in how cycles of poverty and deprivation become entrenched in societies through their adverse impact on educational progress, and thereby on future employment opportunities and earning potential [2, 10]. South Africa is particularly relevant in this regard, as recent evidence on educational outcomes has shown that children are performing exceptionally poorly, even when compared to other LMICs [11]. Notably, there appears to have been a steady worsening over recent years of school grade scores on measures of reading and literacy [12]. Importantly, both cross-sectional and longitudinal research shows that particular parenting practices are associated with child cognitive performance: where parental interactions are responsively contingent to the child, and support the child’s active engagement in their environment, children perform better on measures of cognitive functioning [13, 14].

### Violence and aggression

Aside from cognitive and socioemotional delays, a major problem that disproportionately affects those in LMICs is a high rate of community violence. Indeed, violence is one of the leading causes of premature morbidity and mortality in LMICs, with homicide rates often up to 30 times higher than in HICs [15]. In South Africa, violence has been reported as the second highest contributor to deaths and disability-adjusted life years (DALYs) after HIV/AIDS [16]. Longitudinal evidence shows that the perpetration of violence is predicted by the development of severe and persistent aggression in early childhood [17–19]. Such a trajectory is likely to arise when young children do not acquire adaptive ways of dealing with challenges and frustrations.

Studies in HICs show that aggression in childhood and adolescence is predicted by a range of early child, family, and wider social factors [7, 13, 14]. Key predictors include child concentration problems and hyperactivity, as well as insecure attachment [17]. Importantly, meta-analyses have also revealed the importance of particular parenting problems, namely, the occurrence of harsh and coercive parenting, including corporal punishment, and the lack of parental positive reinforcement and responsiveness, and general insensitivity [20, 21]. Although little is known about parenting and family risk factors for violence in LMICs, the available evidence suggests that the same predictive relationships obtain as in HICs [22, 23].

In addition to evidence concerning the early child and parenting *risks* for later aggression, research has shown a number of early positive child and parenting characteristics to be associated with *reduced* levels of aggression. One key child characteristic is social understanding. This includes the ability to understand one’s own feelings, emotions, desires and intentions, as well as those of other people [24]. A key development in early social understanding is ‘theory of mind’ – the ability to accurately attribute mental states (desires, motives, emotions) to other people and understand that these can be different from one’s own [25]. The development of social understanding is important because it underpins cooperation and prosocial behaviour [24] which, in turn, predict lower rates of aggression and externalising behaviour problems [26, 27].

As with child cognition and aggression, specific aspects of early parenting are important in the development of child social understanding. In particular, research has highlighted the contribution of specific kinds of parental discourse with the child because these are internalised and shape child socioemotional development [28, 29]. When parental speech includes frequent reference to mental states, desires and emotions (e.g. ‘think’, ‘feel’, ‘want’), this helps to promote children’s social understanding, including theory of mind capacity [30, 31].

### Book-sharing

The sharing of picture books with young children is well-established as being of substantial benefit to their language development and preliteracy skills [32–34]. An early meta-analysis of the frequency of parent-child book-sharing on language development, emergent literacy, and reading achievement revealed an overall effect size of *d* = 0.59 on collective language and literacy outcomes [35]. Intervention studies in HICs in which carers have been trained in supportive book-sharing skills have found that this leads to substantial improvements in child cognitive outcomes [36–38]. A more recent meta-analysis assessed the value of dialogic reading interventions and included observational studies, quasi-experiments, and randomised controlled trials [32]. This review showed an overall effect on child vocabulary outcomes of *d* = 0.42; CI 0.30, 0.53. Finally, a recent meta-analysis on the effectiveness of shared picture-book reading interventions on child language outcomes revealed a substantial effect on expressive vocabulary (*d* = 0.57; CI 0.33, 0.81) and a positive, although more modest, effect on receptive language (*d* = 0.26; CI 0.12, 0.41) [39].

Picture books that are rich in mental themes can also be effective tools for prompting mental state discussion between parents and young children [40–42]. Indeed, recent observational research has also shown associations between book-sharing and child social understanding [43, 44]. Thus, there is preliminary evidence that not only is book-sharing a good context in which carers can promote the development of child cognitive skills, but it is also a particularly appropriate context for parents to talk about mental states and thereby promote child social understanding [44].

To date, there is little evidence concerning the benefits of training parents in book-sharing in LIMCs. However, in a randomised trial of a book-sharing intervention we recently conducted in South Africa, with carers of 14–16-month-old infants, we demonstrated substantial gains in infant language and attention [45], and found preliminary evidence of improvements in social understanding and empathy [46]. Notably, mediation analyses showed that it was by virtue of improvements in parental sensitivity and reciprocity that the cognitive gains were brought about [46].

### Current trial

Our previous book-sharing trial [45, 46] focussed on promoting child language and attention. In the current trial the intervention includes a more specific focus on promoting parenting skills relevant to child social understanding and aggressive behaviour. Specifically, the training is extended to include book content with themes concerning book characters’ emotions, intentions, perspectives, and prosocial behaviour, in order to promote parental mental state and emotion talk around these topics. Although we do not directly address the topic of negative parenting in the intervention, we hope to reduce such practices generally as a secondary consequence of promoting positive parenting characteristics in the context of book-sharing.

## Methods

### Study design

**Fig. 1:**
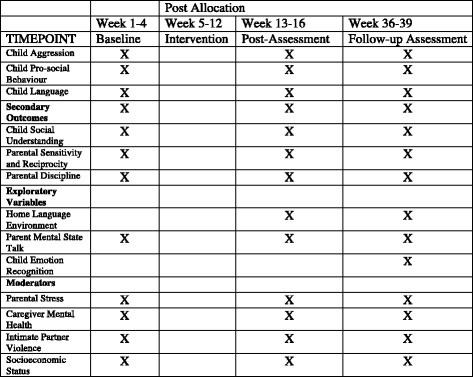
Schedule of enrolment, interventions and assessments. Standard Protocol Items: Recommendations for Interventional Trials (SPIRIT) figure displaying schedule of enrolment, interventions and assessments

We are conducting a randomised controlled trial (RCT) to evaluate a book-sharing intervention for caregivers of children aged between 23 and 27 months designed to promote child cognitive and socioemotional development. Participants are randomised at the individual level to an intervention or a wait-list control group. Data are collected at baseline, post intervention and at follow-up. Figure 1 presents a Standard Protocol Items: Recommendations for Interventional Trials (SPIRIT) representation of the study design. The SPIRIT guidelines for study protocols were followed and a populated SPIRIT checklist is included (see Additional file 1).

Hypotheses:

Primary hypotheses:

1. Compared to control-group children whose carers receive no intervention, those whose carers receive the programme will evidence significantly better outcomes on measures of language and sustained attention
2. Compared to control-group children, intervention-group children will evidence significantly more prosocial behaviour and less aggression

Secondary hypotheses:

3. Compared to control-group children, intervention-group children will evidence significantly better social understanding
4. Compared to control-group caregivers, caregivers who receive the programme will evidence significantly more sensitivity, reciprocity, and mental state talk with their children, both in book-sharing and non-book-sharing contexts
5. Compared to control-group caregivers, caregivers who receive the programme will evidence significantly less negative and more supportive parenting in challenging contexts
6. Improvements in child language and attention will be mediated by improvements in maternal sensitivity and reciprocity in the book-sharing context, and
7. Improvements in child social understanding, prosocial behaviour and aggression will be mediated by increases in maternal sensitivity and mental state talk in both book-sharing and non-book-sharing contexts, and by a reduction in negative parenting and an increase in supportive parenting in challenging contexts

### Collaboration

BEBS is a collaborative project between Stellenbosch University (South Africa), the University of Reading (UK), and Oxford University (UK). The project is funded by the Sexual Violence Research Initiative (SVRI) at the South African Medical Research Council (MRC). Funders have no involvement in the study design or implementation. They may use the published results to write their own reports.

### Study setting

The study is being conducted in Khayelitsha, a large peri-urban township on the outskirts of Cape Town, South Africa. It is characterised by high levels of poverty, unemployment and violence [47]. All assessments take place at the Prevention Research for Community, Family and Child offices at the Masiphulisane Community Development Centre. Intervention group sessions are held in a church hall connected to the Masiphulisane Centre.

### Eligibility criteria

Families who are eligible for inclusion in the study are those with children aged 23–27 months at the time of baseline assessment. They also require an adult primary caregiver who is at least 18 years old, lives in the household with the child for at least four nights per week, and who consents to participate in the study. A chronic illness or disability in the child or the adult that would prevent them from fully participating in the intervention is an exclusion criterion.

### Sample size

A minimum of 140 caregiver-child dyads (70 in each arm) will form the study sample. The sample has considerable power to detect the primary cognitive outcomes and adequate power to detect the primary behavioural ones. The sample size has been calculated using available appropriate effect size estimates on the primary outcomes.

#### Power calculation

All power calculations have been run for 80% power and alpha = 0.05. The power calculation for primary outcomes of language and attention was based on previous research that aimed to improve child language and focal attention through a book-sharing intervention in South Africa [45]. The effect size on sustained attention was *d* = 1.1, and the effect size on receptive language was *d* = 0.73. Based on these figures, two groups of 13 are sufficient to detect the established difference on the attention variable, and two groups of 44 are sufficient to detect the established difference on the language variable. For the behavioural outcomes of aggression and prosocial behaviour, the power estimates were based on normative data from a study in Khayelitsha on a sample of 302 children. For prosocial behaviour the mean was 7.39 (SD = 2.08) and for aggression it was 12.84 (SD = 7.18). As such, for both the prosocial and the aggression variables, two groups of 64 are sufficient to detect the difference (two-tailed), based on an effect size of 0.50. Two groups of 70 are, therefore, being recruited, which allows for 10% sample attrition.

### Recruitment

Recruitment began in February 2016 and ended in August 2016. Participants were recruited from four neighbourhoods within the greater Khayelitsha region. Three of these neighbourhoods fall within the area of Endlovini which consists almost entirely of informal housing structures. One area is located in Makhaza which is characterised by a mix of formal brick housing and informal structures. Recruitment took place in two consecutive waves. Recruiters systematically visited each house in the demarcated area and enquired about potentially eligible participants. Participants who met inclusion criteria and were interested in being part of the study were invited to the research centre.

### Randomisation

Eligible participants who consented to taking part in the study were randomised in a 1:1 ratio to the index group and a wait-list control condition (the latter being offered the intervention once the three waves of assessment have been completed). A minimisation process was used to ensure a similar distribution of participant characteristics between the two study groups in terms of child age (younger (23–25 months) and older (25–27 months) and sex. ND performed the minimisation process, using the MINIM software [48] on site, when participants consented to being part of the study and prior to completion of baseline assessments. ND then notified an isiXhosa-speaking research assistant of the allocation, and this assistant then privately explained the allocation to the participant. The allocation list is held by ND, and is inaccessible to any of the staff or partners involved in the assessment or coding.

### Intervention

The intervention is a group-based, dialogic book-sharing programme based on our previous programme [45, 46, 49]. The intervention consists of 60–90-min sessions run weekly for eight consecutive weeks. The programme is delivered to groups of three to six caregivers and their children. Each session focusses on different and incremental techniques for caregivers to apply during book-sharing. For the first six sessions there is a ‘book of the week’ that the carers take home to share with their child, and that they bring back the following week. In session 7 all the key principles are reviewed, and the child chooses which of the six books they want to take home for that week. During the final, eighth, session, there is a group discussion where caregivers are guided in reflecting on the programme and they discuss plans for continuing with their book-sharing – such as registering at a nearby children’s library or continuing to meet as a group. Session 8 ends with a graduation where caregivers are presented with a certificate of completion, a laminated card with a summary of the key lessons from the programme (on the back of which there is a picture of themselves and their child sharing a book), and a copy of each of the six books used in the programme. Table 1 details the content for each session. For the 6-month period following session 8, the facilitator visits each participant bi-monthly to deliver a new picture book and have a short encouraging conversation with the caregiver about their book-sharing.

**Table 1:** Summary of intervention session content

Session	Session content
1	*Introduction to Book-sharing*
	Introduction and basic dialogic book-sharing skills part 1 – following the child’s lead, using a lively voice, setting up book-sharing routines
2	*Pointing and Naming*
	Basic dialogic book-sharing skills part 2 – pointing and naming, repeating, extending and elaborating on things that interest the child, finding opportunities for praise
3	*Naming and Linking*
	Asking ‘where/who/what’ questions, linking book content to the child’s own experience, finding opportunities to use actions (e.g. hugging, eating)
4	*Talking about Feelings*
	Helping the child understand the meaning of basic emotion terms: happy, sad, angry, scared. Discussing why book characters feel the way they do, using facial expression and tone of voice to convey a character’s feelings, linking feelings to the child’s own experience
5	*Talking about Intentions*
	Discussing why characters feel the way they do, asking what characters are thinking and intending, encouraging the child to be curious about what will come next in the story
6	*Talking about Perspectives*
	Helping the child understand that different people can see things differently, know different things, and feel differently about things
7	*Summary*
	Reviews of key principles from sessions 1–6
8	*Graduation Event*
	Certificates of completion are presented to participants along with summary take-home cards and a full set of the 6 books (from sessions 1–6). A group discussion about how to remain motivated about book-sharing and how to access children’s books (e.g. registering with the local library).

For the intervention sessions, caregivers sit in a semicircle facing the facilitator who sits at the front of the room with a laptop on which a PowerPoint is displayed. An area at the back of the room is laid out for the children with a set of soft toys and balls. The first part of each session is a group-based, instructive presentation of the week’s key book-sharing principles. PowerPoint slides are used to deliver particular learning points, accompanied by brief illustrative video clips. Towards the end of the presentation, the facilitator discusses the book of the week with the carers, highlighting how the book can be used at home and providing examples of how to apply the techniques covered in that session.

The second part of the session involves one-to-one mentoring with the facilitator. This takes place in a separate room, where the caregiver is asked to share the book of the week with their child, under the guidance of the facilitator. These sessions last for approximately 10 min, with the final few minutes dedicated to positive feedback and, where considered helpful,  modelling of book-sharing techniques. This session also includes a discussion between caregiver and facilitator about the book-sharing home routine, where the facilitator encourages the carer to spend at least 10–15 min a day sharing the book of the week with their child and practising the techniques learned that week.

### Intervention facilitators, training and supervision

The intervention is delivered by two isiXhosa-speaking women from Khayelitsha. Both these facilitators have experience delivering book-sharing training to mothers from the community, having been facilitators in our previous trial [45, 46], as well as in an earlier pilot study [49]. Each of the two facilitators is supported by an assistant during intervention sessions whose role is to look after the children, who also attend the sessions. The assistants were selected from the community, based on having previous experience working with young children.

Facilitator training for the current book-sharing intervention was held over a 2-day workshop, run by PC, LM and ND. This included a refresher session, when the content of the earlier programme was reviewed, including a review of the accompanying PowerPoint presentation materials and videos. This was followed by instruction on the new programme content and materials. First, the key principles of the new sessions (i.e. those concerning emotions, intentions and perspectives – see Table 1) were explained, and the accompanying illustrative videos viewed. This was followed by discussion between the trainers and facilitators, and any questions or uncertainties were resolved. During these discussions the facilitators also discussed ways in which the key principles of the new material could be explained and demonstrated in a culturally appropriate manner.

The second part of the facilitator training covered the one-to-one sessions where parents practise book-sharing with their children and receive feedback from the facilitator. This training specified the role that the facilitator needs to play during these sessions, covering principles of sensitive instruction, modelling, and providing positive support. Additionally, facilitators were instructed on how to incorporate into the one-to-one sessions the central take-home messages from the group session.

Finally, facilitators were briefed on monitoring and evaluation procedures, including tracking attendance, scheduling participants for sessions, and following up with participants who miss sessions. They were also given a refresher session on using the PowerPoint presentation materials on laptops. The assistants attended a 1-day training workshop run by ND that covered basic video camera skills, activities to do with the children, and roles and responsibilities during sessions – such as preparing refreshments, filming, watching over the children.

Regular supervision is provided throughout the intervention. The trial manager (ND) meets with the facilitators for 2 to 3 hours at the end of each week and reviews how the sessions that week went. During the first half of the meeting the two facilitators discuss aspects that went well, challenges, and logistical issues. They also identify particular participants who are experiencing difficulties in applying the programme with their child, and discuss the support these individuals may need in their next one-to-one session. The second half of the meeting involves preparation for the following week’s session. The group go through the PowerPoint slides, discuss specific examples or strategies to use, and make sure that the facilitators have no unanswered questions or queries about the forthcoming session. Finally, the group reviews the attendance records from the past week, and discusses plans for catch-up sessions for any participant who may have missed the session. Every intervention session is video-recorded, and one session each week per facilitator is checked by ND for delivery fidelity.

### Data collection

#### Data collector training

A team of three data collectors have been trained in the child assessments and caregiver interview questionnaires. Training was held over a 3-week period and followed a data collector manual developed by ND, LM and PC. All three data collectors had previous experience administering questionnaires and child assessments, including administration of some of the measures used in the present study. During the three assessment waves, ND makes regular checks, both in vivo and though examination of the videos made, to ensure fidelity of assessment administration. All data collectors were familiar with consent and referral procedures, as well as how to discuss potentially sensitive topics during interviews with caregivers.

#### Procedures

All 140 carer/child pairs are assessed on three occasions: at baseline, following the 8-week intervention, and 6 months post intervention. For the baseline assessment, caregivers are contacted by the data collection team, and the study is explained to them, including that participation is entirely voluntary. A suitable time for them to come in for assessments is arranged. A driver picks participants up from their home and brings them into the research centre located in central Khayelitsha. On arrival, participants are offered refreshments for themselves and their child. Consent is then explained again and caregivers provide consent for both themselves and their children. Assessments last for up to 2.5 hours. This comprises specific assessments of the child (e.g. Early Childhood Vigilance Task), interviewing the caregiver (e.g. Communication Development Inventory), and filming the caregiver and child in interactive tasks (e.g. book-sharing). There are frequent breaks for drinks and snacks and, if the child shows any signs of tiredness or distress, the session is interrupted or, if necessary, terminated. At the end of the assessment session participants are given a food voucher (rand equivalent of US$8) for a local grocery store to compensate them for their time. Similar procedures are followed for the subsequent two assessment waves. To minimise assessment bias, assessments of children and caregivers are being carried out by data collectors who are blind to group allocation. All coders of video data will also be blind to allocation. Participants are requested not to reveal their allocation to data collectors.

#### Retention

Provisions have been put in place to maximise participant retention. This includes phone calls to remind participants of scheduled assessments. Where necessary, members of the data collection team visit the participant’s house to discuss arrangements for assessments, making every effort to accommodate the caregiver – including holding assessments on Saturdays for caregivers who work during the week.

### Outcomes

Outcome data are collected through the use of (1) direct child assessments, (2) video-recorded caregiver-child interactions, and (3) caregiver interviews. The caregiver interviews and child assessments used have all been translated into isiXhosa. The assessment of each carer-child pair is completed by two assessors. This comprises assessments of the child (e.g. attention assessment), interviewing the caregiver (e.g. assessing carer depression), and filming the caregiver and child in interactive tasks (e.g. while sharing a book). All outcomes are presented in Table 2.

**Table 2:** Study outcomes. Description of data: list of all study outcomes

Outcome	Measure
Primary outcomes	
Child aggression	Child Behavior Check List (CBCL) aggression subscale
	Scores from coded videos of a prohibition and compliance task
Child prosocial behaviour	Strengths and Difficulties Questionnaire, prosocial subscale
	Prosocial helping task
Child language	MacArthur-Bates Communication Development Inventory
	Peabody Picture Vocabulary Test
	Expressive One Word Picture Vocabulary Test
	Bayley Scale of Language Development
Child attention	Early Child Vigilance Task
Secondary outcomes	
Child social understanding	Wellman and Liu Theory of Mind Scale
	Emotion recognition task
Parental sensitivity and reciprocity	Direct observation of parent and child in free-play, clean-up task, and book-sharing
Parental discipline	Discipline and Violence Self-report Questionnaire
	Direct observation during the prohibition and compliance tasks
Parental mental state talk	Direct observation during interactions involving book-sharing and a narrative cartoon explanation task
Exploratory outcomes	
Home language environment	LENA digital recording device worn by the child for a full day
Emotion recognition	Functional Infared Thermal Imaging (fIRT)
Moderators	
Parental stress	Parenting Stress Index (short form)
Caregiver mental health	Patient Health Questionnaire-9
Intimate partner violence	WHO questionnaire on health and domestic violence

### Primary outcome measures

1. Child cognition

*Child language*

Child language is assessed by interviewing the primary caregiver with the short form of the MacArthur Bates Communication Development Inventory (CDI) [50]. This 100-item checklist, which has previously been used in other African contexts [51], provides information on child expressive and receptive language. Aggregate scores are calculated and used as interval scale variables. This assessment is made at all three assessment points.

An adapted version of the Peabody Picture Vocabulary Test (PPVT) [52] is used to assess child language comprehension directly, at baseline and post intervention. This consists of 24 items displayed on a screen in groups of four. The child is asked to point or ‘touch’ certain items by the assessor (‘Where is the dog. Show me the dog. Point to the dog’).

For expressive language, a 15-item adapted version of the Expressive One Word Picture Vocabulary Test (EOWPVT) [53] is administered at baseline and post intervention. The child is prompted to say the names of images that are displayed on a screen (‘Look at the picture [child’s name]. What is that? What is that called? Can you tell me what that is?’). Furthermore, the Bayley Scale of Infant Development [54] language subscales will be used. These provide a comprehensive measure of expressive and receptive language abilities and have previously been used in a study in peri-urban Kenya [55].

Both the CDI and the adaptation of the Peabody were used in our previous study evaluating book-sharing, and were found to be sensitive to the intervention and to be related to parenting [45, 46, 49].

*Child attention*

Child attention is measured using the Early Childhood Vigilance Task (ECVT) [56]. This is a screen-based assessment of sustained focal attention during which the child views interesting moving cartoon stimuli. The child monitors the screen as images appear, disappear, and then reappear over a period of 7 min. Infant sustained attention is indexed by the number of seconds the child attends to the screen, expressed as a proportion of the 7 min of the video. This assessment is made at all three assessment points. The ECVT has been used successfully in other African contexts [57], as well as in our previous RCT [45, 46].

2. Child behaviour

*Child prosocial behaviour*

This is assessed directly in a prosocial ‘helping’ task where a scenario is created that gives the child the opportunity to help the assessor locate her lost pen [46, 58]. ‘Helping behaviour’ is scored if the child picks up the lost pen and returns it to the assessor, or points to the lost pen, or verbally indicates its location to the assessor. This assessment, based on a task reported by Buttelmann and colleagues [58], was used in our previous trial of book-sharing and was found to show effects of the intervention [46].

In addition, the child’s main caretaker is interviewed using the prosocial scale of the Strengths and Difficulties Questionnaire (SDQ) [59], a measure that has previously been used successfully in South Africa [60]. This is a five-item subscale, that uses a 3-point Likert scale that can be aggregated and used as an interval scale variable of raw scores. This assessment is made at all three assessment points.

*Child aggression*

The child’s main caregiver is interviewed using the aggression subscale of the Child Behavior Checklist (CBCL) for ages 18 months to 5 years [61]. This is a 20-item questionnaire that uses a 3-point Likert scale on which children are rated according to various types of aggressive and defiant behaviour. An aggregate of raw scores is used as an interval scale variable that can be dichotomised. This measure has been used successfully in several South African studies [62–64].

Direct measures of child aggression and defiance/noncompliance are obtained from coded video data of two parent-child interaction tasks: a ‘Don’t touch’ prohibition task, and a ‘Clean up’ compliance task [65–67], and one frustration task – the ‘Barrier’ task. The first two tasks have been used to assess child defiance in several HICs [66, 67]. Since they have, to our knowledge, not been used in LMIC contexts, baseline videos will be examined and culturally sensitive modifications will be made to existing coding schemes. The Barrier task has also been widely used in HICs [68], as well as in our previous work in South Africa [69], to provide a measure of negative reactivity (on the basis of latency (3-point scale) and intensity (5-point scale) of negative emotion), and constructive versus nonconstructive emotion regulation strategy.

### Secondary outcome measures

#### Child social understanding

Child theory of mind is assessed using a set of 10 tasks adapted from the scale developed by Wellman and Liu [70] and used extensively across a range of countries and contexts [25, 30, 70, 71]. The tasks gradually become more complex, beginning with tasks assessing understanding of diverse desires, and moving on to tasks require understanding of diverse beliefs, knowledge access, and false belief. A total score is calculated from the correct answers. This assessment is made at all three assessment points.

Emotion recognition is measured using a set of eight illustrated pictures of children with four different emotional expressions: happy, angry, sad, and frightened. Pictures are presented on a computer screen in blocks of four showing all emotions. The assessor then asks the child to identify certain expressions, e.g. ‘show me the happy boy’. A total score will be calculated from their responses. Emotion recognition and understanding tasks will be administered at the final follow-up. These tasks have been specially designed for the current project, but are based on assessments successfully used by Taumoepeau and Ruffman [72].

#### Parental sensitivity and reciprocity

Parental sensitivity and reciprocity are assessed by direct observation of the parent and child during book-sharing and non-book-sharing contexts. Parent-child interactions are videotaped and independently coded using a reliable system [73], shown in our previous trial to improve with book-sharing training and to mediate the effects of the intervention on child cognitive development [45, 46]. These variables are also rated in the ‘clean-up’ task. All these assessments are made at all three assessment points.

#### Parental discipline

Parenting is assessed using the Discipline and Violence Self-report Questionnaire [74]. This questionnaire comprises 13 items of discipline practice (including violent ones), as well as an assessment of the belief in the necessity of physical punishment in order for children to learn respect for adults. Parental disciplining strategies are also assessed by direct observation of videos of interactions during the prohibition and compliance tasks. Carer behaviour is coded using measures of harsh/coercive discipline (physical and verbal), and supportive guidance (physical and verbal). This assessment of parental discipline has been widely used in the assessment of parenting in several HICs [65, 66]. Since, to our knowledge, they have not been used in a LMIC context, baseline videos will be examined and culturally sensitive modifications will be made to existing coding schemes [66].

### Exploratory outcomes

#### Mental state talk

Parental mental state talk will be assessed by making transcripts (and translating them into English) from video-recordings of interactions during book-sharing, and during a narrative cartoon explanation task. The latter task is one previously used to assess child theory of mind [70, 71]. We show the carer two sets of six cartoon pictures, showing a sequence in which a cartoon character is unaware of another character who is attempting to perform a malevolent act (e.g. a cat attempting to raid a bird’s nest). The carer is asked to tell the child what is happening in the cartoon. Mental state talk will be coded based on a system derived from Ruffman and colleagues [40, 72, 75]. Baseline data will be used to develop a culturally sensitive coding system.

#### Home language environment

In a subsample, the child’s home language environment is assessed using the LENA digital recording system that captures language heard by the child over the course of a typical day. The LENA analysis software produces automated measures of adult word counts that represent the number of words that a child hears from adult men or women during the period of recording. Though predominantly used in HIC contexts, LENA has been used successfully in a low-income context in Senegal [76].

#### Physiological response to emotion

In a subsample, child physiological response to different emotional faces (i.e. pictures of happy, sad, fearful or angry children) is assessed at the pre-intervention and follow up assessment. This is done using a functional infrared thermal imaging (fIRT) camera that converts infrared light into temperature, thereby allowing wireless monitoring of autonomic nervous system responses [77]. We will be recording sympathetic cutaneous reactions to the presentation of images displaying different emotions [78].

### Potential moderators

#### Parental stress

The Parenting Stress Index (short form) [79] is administered. This is a 36-item scale that measures stress associated with the role of parenting that has been used successfully in the South African context [80]. It is administered to the carers at all three assessment time points. In addition, we use, as a potential moderator, an index of socioeconomic adversity, previously found to moderate the impact of a home-visiting intervention in infancy on child cognitive outcome [46].

#### Caregiver mental health

The mental health of the carers is assessed using the Patient Health Questionnaire-9 (PHQ9) [81]. This 10-item questionnaire asks caregivers about depressive symptoms experienced over the previous 2 weeks. It is a multipurpose instrument for screening, diagnosing, and monitoring depression levels, and has been used successfully in previous South African research [82].

#### Intimate partner violence

Caregivers are being questioned on exposure to intimate-partner violence, at all three assessments points, using questions from the measure developed for the WHO multicountry study on women’s health and domestic violence [83].

### Data management

Data are collected on tablets and, once complete, automatically submitted to a secure server platform hosted by Mobenzi, a South African organisation that specialises in software development for research purposes. Participants are allocated personal identification numbers that are used in all study records to protect their identity and maintain confidentiality. All child assessments and caregiver-child interaction tasks are video-recorded, for both coding and quality control purposes. Videos are labelled by ND and saved in a separate location at the end of each day. In addition, a random subsample (10%) of videos is checked by ND for fidelity to the assessment procedure. Data transmitted from the tablets are stored on a secure central network server.

### Data analysis

Data analysis will be completed by a designated statistician/statistical team independent from study investigators. Group baseline differences will be assessed using independent *t* tests and Mann-Whitney *U* nonparametric tests if data do not fit necessary assumptions. Analysis of baseline group differences will also include sociodemographic data such as gender and household factors (e.g. income, employment). Analysis of covariance will be used to assess intervention effects at post intervention and follow-up. Post-test and follow-up scores for primary and secondary outcomes will constitute dependent variables, while child age, sex and baseline scores will be controlled for as covariates. Intention-to-treat analysis will be used to examine intervention effects. Missing individual-level outcome data will be addressed using multiple imputation methods.

#### Mediator analysis

Mediator analysis will aim to identify certain active components of the intervention and elucidate the pathways to change. To this end, the following two questions will be examined: whether improvements in maternal sensitivity and reciprocity in the book-sharing context mediate improvements in child language and attention; and whether increases in maternal sensitivity and mental state talk in both book-sharing and non-book-sharing contexts, and reduction in negative parenting and increase in supportive parenting, mediate improvements in child social understanding, prosocial behaviour, and aggression.

#### Moderator analysis

An exploratory subgroup analysis will be conducted to investigate whether certain groups benefit more or less from the intervention. Subgroup analysis will be explored for carer relationship (mother, father, grandparents), child sex, number of sessions attended, family socioeconomic adversity, parental stress, and caregiver mental health. Potential mediators and moderators of the intervention will be explored using mixed linear models.

### Data monitoring

#### Trial Steering Committee

An independent Trial Steering Committee (TSC) is used to monitor the progress of the trial and advise the research team on matters arising during the course of the study. The TSC meets 6-monthly. The TSC is chaired by a professor of psychology from the University of Cape Town. Its other members, in addition to the principal investigators (PIs) (PJC, LM, MT) and the trial manager (ND), are a representative from a local parenting non-governmental organisation (NGO) (the Parent Centre), a researcher (also from the University of Cape Town), a statistician, and a parent in the area in Khayelitsha where the trial is being conducted, nominated by the Street Committee in Endlovini. If either the assessors or the facilitators detect any indication of negative effects in the children or the carers, they inform the PIs who take appropriate steps to address the problem.

## Discussion

The BEBS trial is an evaluation of an intervention in which carers of young children are provided with training, over 8 weeks, in sharing picture books with their child, following a specific manualised programme. It aims to evaluate the impact of this early parenting intervention on several key risk factors for the development of violence. These include aspects of parenting, in particular low levels of positive, sensitive parenting, and high levels of negative parenting; as well as a number of early child factors, including poor attention, general cognition, and social understanding, and low levels of prosocial behaviour and high levels of aggression. The study is being carried out in a LMIC, South Africa, where violence constitutes a major social and health burden. Since the intervention is brief and, with modest levels of training, readily deliverable in LMIC contexts, a demonstration that it is of benefit to both child cognitive and socioemotional development, would be of significance.

### Dissemination plans

We will use several strategies in our dissemination plan to engage different audiences at national and international levels. This will involve publishing papers in high-impact, open-access (wherever possible), peer-reviewed journals. We will present at appropriate forums such as conferences and invited meetings. We will produce and distribute national and international policy briefs and report the findings of the trial on the website of Prevention Research for Community, Family and Child Health [84]. Finally, we will make the BEBS training materials available through a charity, the Mikhulu Trust [85]. Other, more innovative forms of communication, will also be explored, including social networking sites (e.g. Twitter and Facebook) and blogs linked to the project webpage.

### Trial status

At the point of submitting this manuscript to the journal (29 June 2016) 70/140 (50%) of the sample had been recruited.

## Additional file

Additional file 1: SPIRIT Checklist. Description of data: populated SPIRIT Checklist. (DOC 120 kb)

## Abbreviations

AIDS: Acquired immune deficiency syndrome
BEBS: Benefits of Early Book-Sharing
CBCL: Child Behavior Checklist
CDI: Communication Development Inventory
DALY: Disability-adjusted life years
ECVT: Early Child Vigilance Task
EOWPVT: Expressive One Word Picture Vocabulary Test
fIRT: Functional Infrared Thermal Imaging
HIC: High-income country
HIV: Human immunodeficiency virus
HREC: Health Research Ethics Committee
LMIC: Low- and middle-income country
MRC: Medical Research Council
NGO: Non-governmental organisation
PHQ9: Patient Health Questionnaire-9
PPVT: Peabody Picture Vocabulary Test
PSI: Parenting Stress Index
RCT: Randomised controlled trial
SDQ: Strengths and Difficulties Questionnaire
SVRI: Sexual Violence Research Initiative
TSC: Trial Steering Committee
WHO: World Health Organisation

## References

[1] Grantham-McGregor S, Cheung YB, Cueto S, Glewwe P, Richter L, Strupp B, ICDS Group (2007). Developmental potential in the first 5 years for children in developing countries. Lancet.

[2] Heckman JJ, Masterov DV (2007). The productivity argument for investing in young children. Appl Econ Perspect Policy.

[3] Walker SP, Wachs TD, Grantham-McGregor S, Black MM, Nelson CA, Huffman SL, Baker-Henningham H, Chang SM, Hamadani JD, Lozoff B (2011). Inequality in early childhood: risk and protective factors for early child development. Lancet.

[4] Catts HW, Fey ME, Zhang X, Tomblin JB (2001). Estimating the risk of future reading difficulties in kindergarten children: a research-based model and its clinical implementation. Lang Speech Hear Serv Sch.

[5] Murray L, Arteche A, Fearon P, Halligan S, Croudace T, Cooper P (2010). The effects of maternal postnatal depression and child sex on academic performance at age 16 years: a developmental approach. J Child Psychol Psychiatr.

[6] Bornstein MH, Putnick DL (2012). Cognitive and socioemotional caregiving in developing countries. Child Dev.

[7] Engle PL, Black MM, Behrman JR, De Mello MC, Gertler PJ, Kapiriri L, Martorell R, Young ME, ICDS Group (2007). Strategies to avoid the loss of developmental potential in more than 200 million children in the developing world. Lancet.

[8] Liddell C, Rae G (2001). Predicting early grade retention: a longitudinal investigation of primary school progress in a sample of rural South African children. Br J Educ Psychol.

[9] Dendir S (2014). Children’s cognitive ability, schooling and work: evidence from Ethiopia. Int J Educ Dev.

[10] Heckman JJ. The case for investing in disadvantaged young children. Big Ideas Child. 2008:49–58.

[11] Twist L (2007). Results from PIRLS 2006. Literacy Today.

[12] Modisaotsile BM (2012). The failing standard of basic education in South Africa. Policy Brief.

[13] Moffitt TE, Caspi A (2001). Childhood predictors differentiate life-course persistent and adolescence-limited antisocial pathways among males and females. Dev Psychopathol.

[14] Odgers CL, Caspi A, Russell MA, Sampson RJ, Arsenault L, Moffitt TE (2012). Supportive parenting mediates widening neighborhood socioeconomic disparities in children’s antisocial behavior from ages 5 to 12. Dev Psychopathol.

[15] World Health Organization (2012). World Health Organization global burden of disease.

[16] Seedat M, Van Niekerk A, Jewkes R, Suffla S, Ratele K (2009). Violence and injuries in South Africa: prioritising an agenda for prevention. Lancet.

[17] Tremblay RE, Nagin DS, Seguin JR, Zoccolillo M, Zelazo PD, Boivin M, Perusse D, Japel C (2004). Physical aggression during early childhood: trajectories and predictors. Pediatrics.

[18] Broidy LM, Nagin DS, Tremblay RE, Bates JE, Brame B, Dodge KA, Fergusson D, Horwood JL, Loeber R, Laird R (2003). Developmental trajectories of childhood disruptive behaviors and adolescent delinquency: a six-site, cross-national study. Dev Psychol.

[19] Nagin D, Tremblay RE (1999). Trajectories of boys’ physical aggression, opposition, and hyperactivity on the path to physically violent and nonviolent juvenile delinquency. Child Dev.

[20] Rothbaum F, Weisz JR (1994). Parental caregiving and child externalizing behavior in nonclinical samples: a meta-analysis. Psychol Bull.

[21] Gershoff ET (2002). Corporal punishment by parents and associated child behaviors and experiences: a meta-analytic and theoretical review. Psychol Bull.

[22] Murray J, Anselmi L, Gallo EAG, Fleitlich-Bilyk B, Bordin IA (2013). Epidemiology of childhood conduct problems in Brazil: systematic review and meta-analysis. Soc Psychiatry Psychiatr Epidemiol.

[23] Wessels I, Mikton C, Ward C, Kilbane T, Alves R, Campello G, Madrid B (2013). Preventing violence: evaluating outcomes of parenting programmes.

[24] Ruffman T, Slade L, Crowe E (2002). The relation between children’s and mothers’ mental state language and theory‐of‐mind understanding. Child Dev.

[25] Wellman HM (2002). Understanding the psychological world: developing a theory of mind.

[26] Diener ML, Kim D-Y (2004). Maternal and child predictors of preschool children’s social competence. J Appl Dev Psychol.

[27] Spinrad TL, Stifter CA (2006). Toddlers’ empathy-related responding to distress: predictions from negative emotionality and maternal behavior in infancy. Infancy.

[28] Fivush R, Nelson K (2006). Parent-child reminiscing locates the self in the past. Br J Dev Psychol.

[29] Murray L, Pella JE, De Pascalis L, Arteche A, Pass L, Percy R, Creswell C, Cooper PJ (2014). Socially anxious mothers’ narratives to their children and their relation to child representations and adjustment. Dev Psychopathol.

[30] LaBounty J, Wellman HM, Olson S, Lagattuta K, Liu D (2008). Mothers’ and fathers’ use of internal state talk with their young children. Soc Dev.

[31] Rosnay M, Pons F, Harris PL, Morrell J (2004). A lag between understanding false belief and emotion attribution in young children: relationships with linguistic ability and mothers’ mental-state language. Br J Dev Psychol.

[32] Mol SE, Bus AG, de Jong MT, Smeets DJ (2008). Added value of dialogic parent-child book readings: a meta-analysis. Early Educ Dev.

[33] Lonigan CJ, Shanahan T (2010). Developing early literacy skills: things we know we know and things we know we don’t know. Educ Res.

[34] Whitehurst GJ, Lonigan CJ (1998). Child development and emergent literacy. Child Dev.

[35] Bus AG, Van Ijzendoorn MH, Pellegrini AD (1995). Joint book reading makes for success in learning to read: a meta-analysis on intergenerational transmission of literacy. Rev Educ Res.

[36] Arnold DH, Lonigan CJ, Whitehurst GJ, Epstein JN (1994). Accelerating language development through picture book reading: replication and extension to a videotape training format. J Educ Psychol.

[37] Lonigan CJ, Whitehurst GJ (1998). Relative efficacy of parent and teacher involvement in a shared-reading intervention for preschool children from low-income backgrounds. Early Child Res Q.

[38] Whitehurst GJ, Epstein JN, Angell AL, Payne AC, Crone DA, Fischel JE (1994). Outcomes of an emergent literacy intervention in Head Start. J Educ Psychol.

[39] Dowdall N. The effectiveness of shared picture book reading interventions on child language outcomes: a systematic review and meta-analysis. Master’s Thesis, Oxford University; 2015.

[40] Ruffman T, Slade L, Devitt K, Crowe E (2006). What mothers say and what they do: the relation between parenting, theory of mind, language and conflict/cooperation. Br J Dev Psychol.

[41] Fine Y, Aram D, Ziv M (2014). Promoting low-SES kindergartners’ reference to mental states through parental mediation during interactive storybook reading. Megamot.

[42] Ziv M, Smadja M-L, Aram D (2013). Mothers’ mental-state discourse with preschoolers during storybook reading and wordless storybook telling. Early Child Res Q.

[43] Adrian JE, Clemente RA, Villanueva L (2007). Mothers’ use of cognitive state verbs in picture-book reading and the development of children’s understanding of mind: a longitudinal study. Child Dev.

[44] Adrian JE, Clemente RA, Villanueva L, Rieffe C (2005). Parent-child picture-book reading, mothers’ mental state language and children’s theory of mind. J Child Lang.

[45] Vally Z, Murray L, Tomlinson M, Cooper PJ (2015). The impact of dialogic book‐sharing training on infant language and attention: a randomized controlled trial in a deprived South African community. J Child Psychol Psychiatr.

[46] Murray L, De Pascalis L, Tomlinson M, Vally Z, Dadomo H, MacLachlan B, Woodward C, Cooper PJ. Randomized controlled trial of a book-sharing intervention in a deprived South African community: effects on carer–infant interactions, and their relation to infant cognitive and socioemotional outcome. J Child Psychol Psychiatry. 2016;57:1370-79. 10.1111/jcpp.12605 PMC5659125 27465028

[47] Norman R, Matzopoulos R, Groenewald P, Bradshaw D (2007). The high burden of injuries in South Africa. Bull World Health Organ.

[48] Evans S, Royston P, Day S (2004). Minim: allocation by minimisation in clinical trials.

[49] Cooper P, Vally Z, Cooper H, Sharples A, Radford T, Tomlinson M, Murray L. Promoting mother-infant book-sharing and child cognitive development in an impoverished South African population: a pilot study. Early Child Educ. 2014;42:142-53.

[50] Fenson L, Pethick S, Renda C, Cox JL, Dale PS, Reznick JS. Short-form versions of the MacArthur Communicative Development Inventories. Appl Psycholinguist. 2000;21:95–116.

[51] Law J, Roy P (2008). Parental report of infant language skills: a review of the development and application of the Communicative Development Inventories. Child Adolesc Mental Health.

[52] Dunn DM, Dunn LM. Peabody Picture Vocabulary Test: Manual. Pearson; 2007.

[53] Gardner MF. Expressive one-word picture vocabulary test-revised. Academic Therapy Publications; 1990.

[54] Bayley N. Bayley Scales of Infant Development: manual. Psychological Corporation; 1993.

[55] Sigman M, Neumann C, Jansen AA, Bwibo N. Cognitive abilities of Kenyan children in relation to nutrition, family characteristics, and education. Child Dev. 1989;60:1463–74. 2515042

[56] Goldman DZ, Shapiro EG, Nelson CA (2004). Measurement of vigilance in 2-year-old children. Dev Neuropsychol.

[57] Musielak-Hanold KA. An evaluation of the effects of mediational intervention for sensitizing caregivers (MISC) and a health and nutrition education program on the sustained attention of Ugandan children with HIV. Diss. MICHIGAN STATE UNIVERSITY; 2016.

[58] Buttelmann D, Carpenter M, Tomasello M (2009). Eighteen-month-old infants show false belief understanding in an active helping paradigm. Cognition.

[59] Goodman R (2001). Psychometric properties of the strengths and difficulties questionnaire. J Am Acad Child Adolesc Psychiatry.

[60] Cluver L, Gardner F, Operario D (2007). Psychological distress amongst AIDS‐orphaned children in urban South Africa. J Child Psychol Psychiatr.

[61] Achenbach, Thomas M., and C. Edelbrock. "Child behavior checklist." Burlington (Vt) 7 (1991).

[62] Cluver L, Gardner F (2007). The mental health of children orphaned by AIDS: a review of international and southern African research. J Child Adolesc Mental Health.

[63] Nöthling J, Martin CL, Laughton B, Cotton MF, Seedat S (2013). Maternal post-traumatic stress disorder, depression and alcohol dependence and child behaviour outcomes in mother-child dyads infected with HIV: a longitudinal study. BMJ Open.

[64] Rochat TJ, Houle B, Stein A, Coovadia H, Coutsoudis A, Desmond C, Newell M-L, Bland RM. Exclusive breastfeeding and cognition, executive function and behavioural disorders in primary school-aged children in rural South Africa: a cohort analysis. PLoS Med. 2016:1–51. 10.1371/journal.pmed.1002044 PMC4915617 27328132

[65] Crockenberg S, Litman C (1990). Autonomy as competence in 2-year-olds: maternal correlates of child defiance, compliance, and self-assertion. Dev Psychol.

[66] Kochanska G, Aksan N (1995). Mother‐child mutually positive affect, the quality of child compliance to requests and prohibitions, and maternal control as correlates of early internalization. Child Dev.

[67] Pereira M, Negrão M, Soares I, Mesman J (2014). Decreasing harsh discipline in mothers at risk for maltreatment: a randomized control trial. Infant Mental Health J.

[68] Goldsmith HH, Reilly J, Lemery KS, Longley S, Prescott A. Preschool Laboratory Temperament Assessment Battery (PS Lab-TAB; Version 1.0). Technical Report, Department of Psychology, University of Wisconsin-Madison, 1993.

[69] Bozicevica L, De Pascalis L, Schuitmaker N, Tomlinson M, Cooper P, Murray L. Longitudinal association between child emotion regulation and aggression, and the role of parenting: a comparison of three cultures. Psychopathology. (in press). 10.1159/000447747 PMC5659189 27486811

[70] Wellman HM, Liu D (2004). Scaling of theory‐of‐mind tasks. Child Dev.

[71] Wellman HM, Fang F, Liu D, Zhu L, Liu G (2006). Scaling of theory-of-mind understandings in Chinese children. Psychol Sci.

[72] Taumoepeau M, Ruffman T (2006). Mother and infant talk about mental states relates to desire language and emotion understanding. Child Dev.

[73] Murray L, Stanley C, Hooper R, King F, Fiori‐Cowley A (1996). The role of infant factors in postnatal depression and mother‐infant interactions. Dev Med Child Neurol.

[74] Lansford JE, Deater‐Deckard K (2012). Childrearing discipline and violence in developing countries. Child Dev.

[75] Taumoepeau M, Ruffman T (2008). Stepping stones to others’ minds: maternal talk relates to child mental state language and emotion understanding at 15, 24, and 33 months. Child Dev.

[76] Weber A, Fernald A, Yatma D. When cultural norms discourage talking to babies: effectiveness of a parenting program in rural Senegal. Child Dev. (in press). 10.1111/cdev.12882 28650107

[77] Ring E, Ammer K (2012). Infrared thermal imaging in medicine. Physiol Meas.

[78] Merla A, Di Donato L, Rossini P, Romani G (2004). Emotion detection through functional infrared imaging: preliminary results. Biomed Tech.

[79] Abidin RR (1990). Parenting Stress Index-short form.

[80] Rochat TJ, Arteche AX, Stein A, Mitchell J, Bland RM (2015). Maternal and child psychological outcomes of HIV disclosure to young children in rural South Africa: the Amagugu intervention. AIDS.

[81] Löwe B, Kroenke K, Herzog W, Gräfe K (2004). Measuring depression outcome with a brief self-report instrument: sensitivity to change of the Patient Health Questionnaire (PHQ-9). J Affect Disord.

[82] Bhana A, Rathod SD, Selohilwe O, Kathree T, Petersen I (2015). The validity of the Patient Health Questionnaire for screening depression in chronic care patients in primary health care in South Africa. BMC Psychiatry.

[83] García-Moreno C, Jansen HA, Ellsberg M, Heise L, Watts C. WHO multi-country study on women’s health and domestic violence against women: initial results on prevalence, health outcomes and women’s responses. World Health Organization; 2005.

[84] Prevention Research for Community Family and Child Health. http://www.preventionresearch.org.za.

[85] The Mikhulu Trust. http://www.mikhulutrust.org.

